# Psychiatric Comorbidities, Legal Considerations and Management of the Incel Phenomenon: A Scoping Review

**DOI:** 10.1192/j.eurpsy.2026.11278

**Published:** 2026-07-31

**Authors:** V. Sharma, R. K. Suri, T. Kainth, S. Prasad, M. Rocha, G. Gill, A. Khadivi, S. Gunturu

**Affiliations:** 1 Dayanand Medical College & Hospital, Ludhiana, India; 2 Psychiatry, BronxCare Health System, The Bronx, United States; 3 American University of the Caribbean School of Medicine, Cupecoy, Sint Maarten (Dutch Part); 4 Psychiatry, Case Western Reserve University/University Hospitals, Cleveland , United States

## Abstract

**Introduction:**

Incels, or ‘involuntary celibates,’ are self-identified individuals who desire sexual or romantic relationships but struggle to find willing partners. This phenomenon is characterized by imposed social isolation and mental health morbidity manifesting as misogynist views, participation in online hate groups and in some cases, as aggressive actions.

**Objectives:**

This scoping review aims to understand the associated attitudes and behaviors, psychiatric comorbidities, and risk factors to optimally screen and manage patients with incel ideology who present to mental health clinics. It also explores the link between inceldom and violent tendencies, with a special focus on the mass shootings/murders caused by individuals who identified as incels.

**Methods:**

We searched PubMed, PsycINFO, and Scopus using the keyword “incel,” yielding 310 studies. Exclusion criteria included non-English, irrelevant, or inaccessible studies. After removing duplicates (84 articles), we conducted abstract screening and full-text analysis, resulting in 46 studies being included as primary sources. Additional sources from: 1) open-source Google searches spanning incel forums, news articles, and blogs, and 2) references from primary sources increased the total to 124 studies (26 studies on incel linguistics and beliefs, 27 studies on history and evolution, 36 studies on incel violence, 23 studies on biopsychosocial pathophysiology, and 19 studies on psychiatric comorbidities). PRISMA-ScR guidelines were followed (Figure 1).

**Results:**

Incels are primarily young, socially isolated males who face economic and social challenges. They often exhibit significant psychiatric comorbidities—such as depression, anxiety, personality disorders, and suicidality—which, along with social influences, may exacerbate their propensity for violence targeting women and “normies” online and through mass homicidal incidents. Clinical management of incels includes an empathetic understanding of their misogynistic ideologies, pharmacological treatment for underlying psychiatric conditions, psychotherapy to combat extremist beliefs, and forensic assessment for trial competency.

**Image 1:**
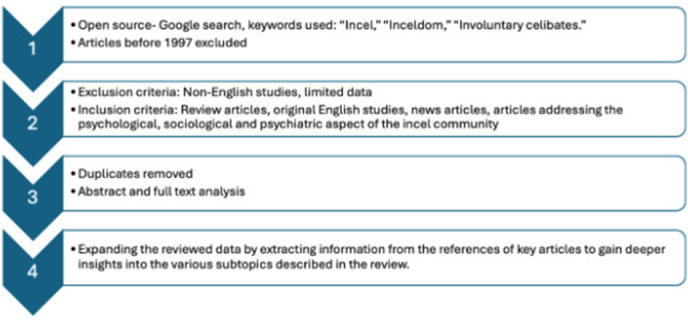
Image 1: Long description.

**Conclusions:**

A comprehensive and multifaceted approach that includes integrated psychiatric care, legal evaluation, and targeted interventions is needed to address violence, psychiatric disorders, and extremist beliefs among Incels.

**Disclosure of Interest:**

None Declared

## Long descriptions

### Image 1: Long description

The flowchart consists of four horizontal rounded rectangles, each numbered on the left within a blue chevron arrow pointing downward to the next step.

* Step 1. Open source- Google search, keywords used: Incel, Inceldom, Involuntary celibates. Articles before 1997 excluded.

* Step 2. Exclusion criteria: Non-English studies, limited data. Inclusion criteria: Review articles, original English studies, news articles, articles addressing the psychological, sociological and psychiatric aspect of the incel community.

* Step 3. Duplicates removed. Abstract and full text analysis.

* Step 4. Expanding the reviewed data by extracting information from the references of key articles to gain deeper insights into the various subtopics described in the review.

Navigate back to Image 1:.

